# Supplementary material for: Causal Relationship between Aging and Anorexia Nervosa: A White-Matter-Microstructure-Mediated Mendelian Randomization Analysis
Source: Biomedicines. 2024 Aug 16;12(8):1874. doi: 10.3390/biomedicines12081874 (PMC11351342; doi:10.3390/biomedicines12081874)
Supplement: Supplementary file 1 [file biomedicines-12-01874-s001.zip › Supplementary Figure/Figure S7/Single SNP plot of MR analysis for ubm-b-1515 to AN.pdf]

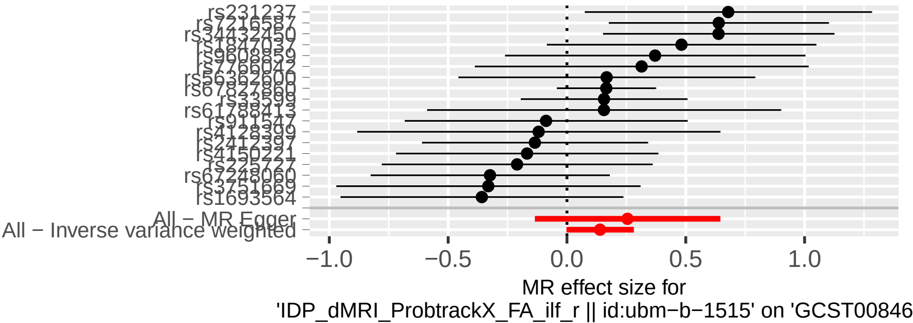
rs231237
rs7216587
rs34432450
rs1847037
rs9608859
rs7766042
rs56362600
rs67827860
rs33599
rs61788413
rs9111547
rs4128399
rs2412397
rs4150221
rs225727
rs67248060
rs3751669
rs1693564
All − MR Egger
All − Inverse variance weighted
−1.0
−0.5
0.0
0.5
1.0
MR effect size for
'IDP_dMRI_ProbtrackX_FA_ilf_r || id:ubm−b−1515' on 'GCST00846
